# HIV testing and the care continuum among transgender women: population estimates from Rio de Janeiro, Brazil

**DOI:** 10.7448/IAS.20.1.21873

**Published:** 2017-09-19

**Authors:** Emilia M. Jalil, Erin C. Wilson, Paula M. Luz, Luciane Velasque, Ronaldo I. Moreira, Cristiane V. Castro, Laylla Monteiro, Ana Cristina F. Garcia, Sandra W. Cardoso, Lara E. Coelho, Willi McFarland, Albert Y. Liu, Valdilea G. Veloso, Susan Buchbinder, Beatriz Grinsztejn

**Affiliations:** ^a^ Evandro Chagas National Institute of Infectious Diseases, Oswaldo Cruz Foundation, Rio de Janeiro, Brazil; ^b^ Bridge HIV, San Francisco Department of Public Health, San Francisco, CA, USA; ^c^ Departamento de Matemática e Estatística, Universidade Federal do Estado do Rio de Janeiro, Rio de Janeiro, Brazil; ^d^ Departments of Medicine, Epidemiology and Biostatistics, University of California, San Francisco, San Francisco, CA, USA

**Keywords:** HIV, antiretroviral therapy, care continuum, transgender women, HIV testing, Brazil

## Abstract

**Introduction**: Evidence suggests that, of all affected populations, transgender women (transwomen) may have the heaviest HIV burden worldwide. Little is known about HIV linkage and care outcomes for transwomen. We aimed to estimate population-level indicators of the HIV cascade of care continuum, and to evaluate factors associated with viral suppression among transwomen in Rio de Janeiro, Brazil.

**Methods**: We conducted a respondent-driven sampling (RDS) study of transwomen from August 2015 to January 2016 in Rio de Janeiro, Brazil and collected data on linkage and access to care, antiretroviral treatment and performed HIV viral load testing. We derived population-based estimates of cascade indicators using sampling weights and conducted RDS-weighted logistic regression analyses to evaluate correlates of viral suppression (viral load ≤50 copies/mL).

**Results**: Of the 345 transwomen included in the study, 89.2% (95% CI 55–100%) had been previously tested for HIV, 77.5% (95% CI 48.7–100%) had been previously diagnosed with HIV, 67.2% (95% CI 39.2–95.2) reported linkage to care, 62.2% (95% CI 35.4–88.9) were currently on ART and 35.4% (95% CI 9.5–61.4%) had an undetectable viral load. The final adjusted RDS-weighted logistic regression model for viral suppression indicated that those who self-identified as black (adjusted odds ratio [aOR] 0.06, 95% CI 0.01–0.53, *p* < 0.01), reported earning ≤U$160/month (aOR 0.11, 95% CI 0.16–0.87, *p* = 0.04) or reported unstable housing (aOR 0.08, 95% CI 0.01–0.43, *p* < 0.01) had significantly lower odds of viral suppression.

**Conclusions**: Our cascade indicators for transwomen showed modest ART use and low viral suppression rates. Multi-level efforts including gender affirming care provision are urgently needed to decrease disparities in HIV clinical outcomes among transwomen and reduce secondary HIV transmission to their partners.

## Introduction

Of all populations affected by HIV, evidence suggests that transgender women (transwomen) may have the heaviest HIV burden worldwide [1]. The rate of HIV infection among transwomen is higher than other at high risk groups, yet most tracking systems do not record data on trans people systematically [2,3]. Measurement of the HIV care cascade has played an important role in monitoring progress in test and treat strategies to curb the HIV epidemic and achieve the 90–90–90 goals [4]. The HIV cascade (or continuum) was developed by Gardner et al. to measure HIV testing, linkage and retention in HIV care and viral suppression [5]. However, little is known about HIV linkage and care outcomes for transwomen, in part, because data about trans women are usually grouped together with men who have sex with men (MSM). For example, a recently published study from Peru found low HIV status awareness, retention in care, uptake of ART and virologic suppression among transwomen and MSM [6].

Disaggregated data from San Francisco show that transwomen have the lowest survival rates compared to men, the lowest insurance rates and have lower rates of linkage to care [3]. Viral suppression is also considerably lower for transwomen living with HIV compared to all other demographic groups except for people who inject drugs [3]. An analysis of HIV surveillance data from New York during 2011–2016 found that compared to MSM, transwomen had increased odds of not achieving viral suppression [7].

In low- and middle-income countries (LMIC) with HIV epidemics concentrated among key populations very little is known about transwomen’s HIV treatment outcomes. As a result, transwomen remain underserved, and services for this population remain under-resourced. The aims of this study were to estimate population-level indicators of the HIV cascade of care continuum, from testing to linkage to care, treatment and viral suppression, and to evaluate factors associated with viral suppression among transwomen in Rio de Janeiro, Brazil.

## Methods

To determine estimates of the HIV cascade of care continuum among transwomen in Brazil, we conducted a secondary analysis of data from *Transcender*, a respondent-driven sampling (RDS) study of 345 transwomen conducted from August 2015 to January 2016 at the Oswaldo Cruz Foundation, in Rio de Janeiro, Brazil. To participate, individuals had to self-identify as a transwoman, living in Rio de Janeiro or the metropolitan area, and be age 18 years or older. Details of the study methodology have been described elsewhere [8]. In brief, based on formative focus group findings, twelve seeds were selected to ensure that the sample was not over-represented by key variables, e.g. age, race/skin colour, trans identities, education, geography, HIV status, history of sex work, and risk behaviours. Participants received up to five coupons that were used to refer peers to the study until the sample size was reached, and equilibrium was achieved on key variables. Equilibrium was reached when the sample composition from one wave to the next differed by less than 2% in the variables education, geography, race/ethnicity, sex work status and HIV status [9]. The present analysis included 141 transwomen living with HIV, of which 101 had a recognized HIV infection at the time of study participation and 40 did not.

Recruitment was completed in 26 weeks with a mean of 3.6 recruitment waves for active seeds (standard deviation [SD] 1.6). Incentives for study participation included snacks, sexual health materials, make up, and a medical visit scheduled for after study enrolment. The coupon return rate was 37%. Socio-demographic data included age, race/ethnicity, schooling, income, drug use and other information related to health and access to care. Self-report of HIV serostatus awareness, access to and linkage to care (defined as attending at least one HIV-medical appointment) and current use of antiretroviral treatment (ART) were also collected. HIV viral load and CD4 lymphocyte count assessments were performed. All 345 participants, regardless of self-reported HIV serostatus, were offered an HIV test that 99% accepted; HIV testing was performed following the Brazilian Ministry of Health algorithm [10]. All participants with a positive HIV test had their HIV viral load (VL) (Real Time PCR) and CD4+ cell count assessed. Participants who tested positive for HIV and were unaware of their serostatus or were not linked to care were immediately offered linkage to specialized HIV care and ART.

We calculated population-based estimates of the HIV care cascade using the RDSII estimator to weight and adjust population estimates according to the recruitment design. In addition, RDS-weighted logistic regression models were used to quantify the association of independent variables with viral suppression (VL <50 copies/ml) among those with known HIV infection. For the regression model, we excluded participants who were unaware of their HIV infection (*n* = 40), leaving us with data from 101 transwomen living with HIV. All the remaining analyses were done for all 141 transwomen. Variables significantly associated with the outcome using a 0.2 significance threshold were included in the initial adjusted model except for linkage to care, which was forced in the model; the final adjusted model retained only the significant variables. All analysis considered statistical significance of 5% and were performed with the RDS Analyst Software version 0.57 [11].

## Ethical aspects

The Evandro Chagas National Institute of Infectious Diseases-FIOCRUZ Institutional Review Board provided ethical approval for this project. All participants signed informed consent forms before study procedures began. This study was sponsored by the Brazilian Research Council (CNPq) and the National Institute of Allergy and Infectious Diseases (NIAID-NIH).

## Results

Table 1 summarizes the characteristics of transwomen living with HIV. Most participants were 35 years of age or younger, self-identified as mixed race or black (80.5%) and had a very low monthly income (54.9%). Roughly 12% had less than 4 years of formal education and only 1.6% had more than a high school education. Very few had access to trans-specific health care (12.4%) or had a transgender surgery (1.7%). The majority of transwomen living with HIV had exchanged sex for money over their lifetime (85.8%), and almost 70% were currently doing sex work. Unstable housing was reported by 56.4%. High frequency of illicit drug use in the prior 12 months was also reported (75%).

**Table 1.** Socio-demographic, behavioural and clinical characteristics of transwomen living with HIV, Rio de Janeiro, Brazil

Variable	RDS weighted, % (95% CI)
**Age (years)**	
18–24	10.1 (0.0–26.7)
25–35	50.8 (27.6–74.0)
36–45	22.1 (9.7–34.6)
>45	17.0 (0.0–42.2)
**Self-declared race/skin colour**	
White	19.5 (4.7–34.2)
Mixed/other	39.3 (16.5–62.1)
Black	41.2 (13.8–68.6)
**Monthly income (in R$)**	
≤500 (160 USD)	54.9 (30–79.6)
501–1000 (160–320 USD)	33.6 (7.6–59.7)
>1000 (320 USD)	11.5 (0.0–24.6)
**Years of formal education**	
<4	11.6 (0.0–25.8)
4–8	27.8 (12.7–42.9)
9–12	59.0 (52.7–65.3)
>12	1.6 (0.0–20.1)
**Gender identity**	
Travesti	36.8 (21.3–52.4)
Woman	24.3 (8.2–40.4)
Transgender woman	37.2 (15.3–59.2)
Other definitions	1.6 (0.0–14.1)
**Lack of access to healthcare due to self-identification as trans**	21.8 (12.1–31.5)
**Access to trans-specific healthcare**	12.4 (5.3–19.6)
**Ever had transgender surgeries**	1.7 (0.0–7.3)
**Housing instability**	56.4 (30.8–81.9)
**Sex work (ever)**	85.8 (69.4–100)
**Sex work (current)**	68.7 (39.5–98.0)
**Illicit drug use in the prior 12 mo^1^**	75.0 (56.3–93.8)
**Binge drinking**	69.9 (53.2–86.6)
**CD4+ cell count (cells/mm^3^)**	
≤200	8.4 (0.0–29.4)
201–350	4.5 (0.0–15.6)
351–500	13.7 (0.0–29.0)
>500	73.3 (55.9–90.7)

RDS: respondent-driven sampling; CI: confidence interval

**Figure 1.**
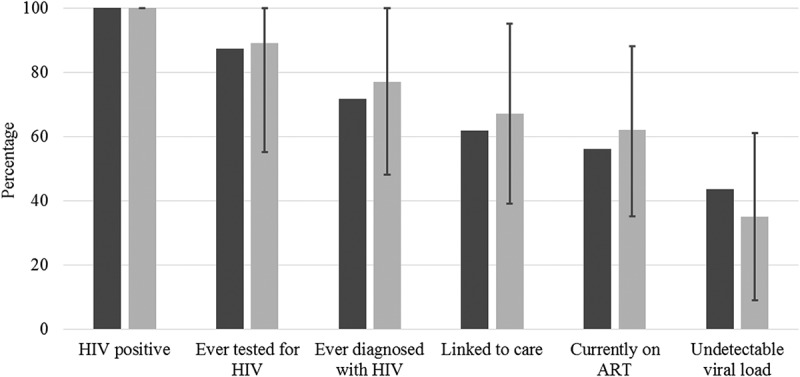
The HIV care continuum among HIV-positive transgender women in Rio de Janeiro, Brazil (*N* = 141). Crude percentages in dark grey, respondent-driven sampling weighted population estimates in light grey, error bars represent 95% confidence intervals for population estimates. * *N* = 138 for denominator with undetectable viral load due to missing data.

As for the cascade indicators (Figure 1), 89.2% (95% confidence interval [95%CI] 55–100%) had been previously tested for HIV, 77.5% (95% CI 48.7–100%) had been previously diagnosed with HIV and 67.2% (95% CI 39.2–95.2) reported linkage to care. In addition, 62.2% (95% CI 35.4–88.9) were currently on ART and 35.4% (95% CI 9.5–61.4%) had an undetectable viral load.

Among those aware of their HIV infection (*N* = 101), 80.0% (95% CI 55–100%) were on ART, 45.0% (95% CI 20–70%) had an undetectable viral load and the median CD4 was 695 cells/mm^3^ (IQR: 467–946 cells/mm^3^). Among those aware of their HIV infection but who were not on ART (*N* = 22), the median CD4 was 398 cells/mm^3^; nine (41%) were not linked to care. Median RDS-weighted duration of ART use was 2.1 years (interquartile range 0.6–8.7 years) with 41 (52%) reporting use of non-nucleoside reverse transcriptase inhibitor-based regimens (most frequent was tenofovir, lamivudine and efavirenz) and 26 (33%) reporting use of protease inhibitor-based regimens (most frequent was tenofovir, lamivudine and boosted atazanavir).

The final adjusted RDS-weighted logistic regression model for viral suppression indicated that race/skin colour, income, housing instability and linkage to care were associated with viral suppression. Participants who self-identified as black (adjusted odds ratio [aOR] 0.06, 95% CI 0.01–0.53, *p* < 0.01) compared to white, reported earning ≤U$160/month (aOR 0.11, 95% CI 0.16–0.87, *p* = 0.04) compared to >U$160/month, or reported unstable housing (aOR 0.08, 95% CI 0.01–0.43, *p* < 0.01) had significantly lower odds of viral suppression. In contrast, transwomen reporting linkage to care (i.e., defined as ever attending an HIV medical appointment) had a borderline significant higher odds of viral suppression (aOR 3.56, 95% CI 0.74–17.2, *p* = 0.11).

## Discussion

This analysis points to suboptimal HIV care linkage, ART use and viral suppression among transwomen living with HIV in Brazil. Our findings show that just over a third of transwomen living with HIV were virally suppressed. When comparing our results to the Brazilian national estimates of the HIV care continuum, ART coverage was quite similar to all Brazilians living with HIV (62.2% among transwomen vs. 52% among Brazilians overall) [12], but viral suppression among transwomen was lower (35.4% for transwomen vs. 46% for Brazilians overall). Lower viral suppression among transwomen compared to other gender groups or risk categories was also reported in a RDS study of transwomen in San Francisco [13], but contrasts with findings from a retrospective cohort study that showed similar treatment outcomes between transgender and non-transgender persons living with HIV [14]. However, the latter study recruited a convenience sample of participants from 13 HIV clinics that were part of an HIV research network and likely had strong retention and adherence strategies in place.

Our findings highlight the relationship between social determinants and health and are consistent with the literature. We found that black racial identity, low income and unstable housing were associated with significantly lower odds of viral suppression. In the study overall (i.e., *Transcender)* and a recent study of MSM in Rio de Janeiro, newly diagnosed HIV infection was associated with non-white race [8,15]. And research from San Francisco has shown that housing instability [13] and low income [16] is associated with poor viral suppression. Socio-economic inequalities, including housing instability, will need to be addressed to improve HIV care linkage, ART use and adherence and rates of viral suppression among transwomen living with HIV in Brazil.

We also found that linkage to care was significantly associated with increased odds of viral suppression. However, few (12%) transwomen in our study had access to trans-specific healthcare, and it is known that transwomen have significant challenges to accessing primary and HIV medical care, even where it is available [13,17,18]. Additionally, studies of transwomen living with HIV in the USA and Canada identified fear of disclosure of transgender identity, poor treatment by staff, and providers’ lack of awareness of transgender issues, as important barriers to engagement in care [19,20]. Lack of access or adherence can result in late presentation (or no presentation) to HIV medical care [21], and overall poor health outcomes among transwomen living with HIV. Our findings point to barriers to healthcare access among transwomen. Gender affirming care includes the correct use of pronouns and provision of hormone therapy, among others, and may be offered even in settings without referral centres specialized in transgender healthcare. Such services have been shown to reduce mental health risks [22,23] and improve quality of life for transgender people [24]. In the absence of the ability of the public health system to end systemic discrimination towards transwomen, offering hormones and providing an environment where transwomen’s gender identity is respected may be one way to improve linkage to care and increase HIV viral suppression among transwomen. Interventions to better provide medical care for transwomen may affect HIV disease progression within the population, which also have implications for onward HIV transmission [25] and research to best capture the HIV care continuum among transwomen.

Our study is not without limitations. This is a cross-sectional study, which limits temporal analysis and determination of the directionality of association. RDS-weighted estimates provide population-based data; yet, there is an ongoing debate as to which estimators are the most accurate for use, and should therefore be reviewed with caution. While our findings may not generalize to all transwomen in Brazil, they suggest that transwomen have similar rates of ART coverage as non-transgender men when engaged in care. A major strength of this study is that estimates of the HIV care continuum were biologically confirmed and show low viral suppression among transwomen, pointing to next steps in HIV care interventions.

## Conclusions

HIV risk remains very high among transwomen, emphasizing the urgent need for prevention interventions such as pre-exposure prophylaxis and others. Our results emphasize structural vulnerabilities as key factors impeding viral suppression. Better healthcare access is needed in Brazil to address the persistent and growing HIV epidemic among transwomen. Our findings also point to the inequalities in viral suppression for transwomen compared to other key populations, which serve to continue to fuel the HIV epidemic in Brazil. Interventions to improve linkage and engagement to HIV treatment and care for transwomen will help transwomen achieve viral suppression and address inequalities resulting in disparities along the HIV care cascade.
